# Safety of multi-access site venous closure following catheter ablation of atrial fibrillation and flutter

**DOI:** 10.1007/s10840-024-01773-3

**Published:** 2024-02-27

**Authors:** Sanjaya Gupta, Raghu Kolluri, Tiessa Simoes, Sandeep C. Pingle, Hong Nie, Michael S. Lloyd, Daniel Steinhaus, Stacy B. Westerman, Anand Shah, Jessica Kline, Soroosh Kiani

**Affiliations:** 1 Division of Cardiology, Saint Luke’s Mid-America Heart Institute, 9th Floor, Cardiovascular Research, Kansas City, MO 64111 USA; 2 University of Missouri Kansas City School of Medicine, Kansas City, MO USA; 3 Syntropic Core Lab, Columbus, OH USA; 4 Abbott Vascular, Santa Clara, CA USA; 5 Division of Cardiology, Emory University, Atlanta, GA USA; 6 Division of Cardiovascular Medicine, UMass Chan Medical School, Worcester, MA USA

**Keywords:** Closure device, Catheter ablation, Atrial fibrillation, Duplex ultrasound

## Abstract

**Background:**

Following catheter ablation, vascular access management involves potential complications and prolonged recovery. Recently, suture-mediated closure (SMC) devices were approved for venous access procedures. The objective of this study is to evaluate the safety of a commercially available SMC for multiple access site venous closure by duplex ultrasound (DUS) in asymptomatic subjects with non-visible complications.

**Methods:**

Thirty-six subjects (63 ± 10.7 years old, 12 female) were enrolled. Following catheter ablation for atrial fibrillation, all subjects had SMC of every venous access site. Subjects underwent DUS of femoral veins and arteries. DUS was performed at discharge, and again at 30 days. Subjects were evaluated for clinically apparent vascular complications.

**Results:**

Mean procedure duration was 138.6 min, and the time to hemostasis was 3.1 min/access site and 9.5 min/subject. Median time to ambulation was 193.5 min, and median time to discharge was 5.95 h, with discharge as early as 2.4 h. A median of 2 sheaths/vein and a median of 2 SMC devices/vein were used. There were no major complications and a 16.7% (6/36) minor complication rate at discharge. All complications resolved at 30 days. The complication rate was not higher in patients with 2 SMC per access site as compared to the patients who just received 1 SMC per access site.

**Conclusions:**

This study demonstrates the safety of multi-access closure using SMC, following catheter ablation procedures, for closure of sites that use sheath sizes from ≤ 8F to ≥ 15F and for those that use 2 or more SMCs per access site.

**Graphical abstract:**

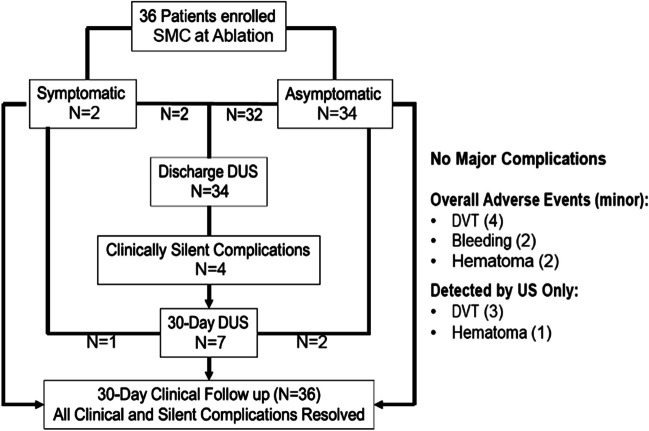

## Introduction

Catheter ablation of atrial fibrillation and atrial flutter involves obtaining vascular access in the right and/or left common femoral veins. Depending upon the number of catheters involved in the procedure, the number of access sites may range from 1 to 3 sites per vein. At the conclusion of the procedure, once catheters are removed, the access sites are closed, and the patient is transferred to recovery. The process of vessel closure to obtain hemostasis has evolved from manual pressure to a subcuticular purse-string suture (i.e., “figure-of-8” stitch) to various forms of closure devices [1–5]. Closure devices are established to be safe and effective for arterial closure for diagnostic and interventional procedures. Vascular access closure with suture-mediated closure (SMC) has been approved for use in venous closure based on data from structural interventional procedures. This procedure involved the insertion of a single large-bore venous sheath, and therefore, access site closure was only studied at a single access site [6]. Venous vascular access closure with SMC at multiple sites has been studied in a registry in comparison to manual pressure and the use of a figure-of-8 stitch [7–9]. All of these studies involved only clinical assessment of outcomes and adverse events. However, no study to date has examined multi-site SMC of venous vascular access using duplex ultrasound (DUS) evaluation with endpoints independently adjudicated.

The objective of this study is to evaluate the safety and efficacy of multiple access site closure with SMC for patients undergoing catheter ablation for atrial fibrillation and flutter. The safety evaluation is by scheduled DUS pre-discharge and at 30 days for patients with abnormal findings adjudicated by an independent core laboratory.

## Methods

This is a prospective, multi-center, single-arm cohort study. The study was performed at two centers with experience in both catheter ablation and the use of vascular access site closure devices. The study was registered at www.clinicaltrials.gov under identified NCT04904809. All subjects gave their informed consent for inclusion before they participated in the study. Only one patient who was screened for inclusion in the study declined participation; the remainder of consecutively screened patients agreed to participate in this study. The study was conducted in accordance with the Declaration of Helsinki, and the protocol was approved by the Institutional Review Board of Saint Luke’s Health System. The inclusion criteria for this study were patients aged 18 years or older, who were scheduled to undergo catheter ablation procedure with planned multiple access sites in a single femoral vein, all access sites planned to be closed with Perclose™ ProGlide™ and Perclose™ ProStyle™ SMC (Abbott Cardiovascular, Plymouth, MN), and written informed consent obtained prior to the procedure. Exclusion criteria included visible thrombus on ultrasound or angiogram prior to the ablation procedure, prior ipsilateral deep vein thrombosis (DVT), INR > 3.5, pregnancy, patients unable to ambulate pre-procedure, and patients with symptoms consistent with COVID-19 infection (due to potential increased risk of a vascular thrombosis seen in these patients) and/or a positive test. Consecutive cases were included with no patients excluded due to operator preference or judgement regarding closure method. All seven operators were highly experienced in use of SMC.

The primary endpoint of this study was vascular complications detected by scheduled DUS at discharge or repeat DUS at 30 days post-discharge in patients with either asymptomatic or non-visible complications at discharge. The specific complications assessed were pseudoaneurysm, hematoma, deep venous thrombosis, arteriovenous fistula, dissection, stenosis > 50%, arterial tear/perforation with need for vascular surgery, and excessive bleeding requiring transfusion or prolonged manual pressure to achieve hemostasis. Secondary endpoints were any vascular and access site complications.

For patients on a direct oral anticoagulant, one dose of medication was held on the morning of the procedure, whereas patients on warfarin did not hold medication but were verified to have an INR between 2 and 3. All patients had vascular access using the modified Seldinger technique. The use of vascular ultrasound to guide access was routinely used. For multiple access sites in a single vessel, vascular access was obtained from caudal to cranial location with larger sheaths being placed in the cranial position. After vascular access was obtained, a pre-close technique was used to deploy the SMC for all access sites in all patients. A single SMC was used for all sheath sizes of 10Fr or less. For sheath sizes greater than 12Fr, an additional SMC was used if there was concern for additional bleeding, based upon operator experience. For patients undergoing two SMC in the same vessel, the SMC device was rotated approximately 45° to facilitate deployment of suture in orthogonal directions to facilitate large vessel closure. Once all vascular access was obtained, intravenous heparin was given for all patients undergoing an ablation in the left atrium. Heparin was given in bolus and continuous infusion to target an ACT of 280–320 s. At the conclusion of the procedure, all access sites were closed using the standard SMC technique that has been described previously [10]. The use of protamine post-procedure was used according to operator discretion. Manual pressure and/or lidocaine with epinephrine injection were routinely performed to address any superficial oozing at the access site. Patients were allowed to ambulate at 2 h post-procedure per protocol, but this duration varied at the discretion of the individual operator’s preference as well as the nursing assessment of patient’s recovery from anesthesia. Anticoagulant medication was resumed the evening of the procedure at the usual prescribed time.

All suspected complications were assessed by clinical staff and documented as related or unrelated to the procedure. All adverse events were adjudicated by a clinical events committee (CEC). Major complications were defined as those which require surgical, interventional, or pre-specified repair and/or hospitalization. All other complications were considered minor complications. DUS was scheduled at discharge in all patients. A 30-day DUS was performed in patients with vascular complications at discharge. The primary endpoints (major and minor) were adjudicated by the duplex core lab. The total duration of the study was 6 months.

The core lab recommended DUS protocols were followed at the enrolling sites. The access site veins were assessed for compressibility, flow, and the presence of deep vein thrombosis, stenosis > 50%, venous tear, or venous perforation. The corresponding arteries were examined for signs of pseudoaneurysm, AV fistula, dissection, stenosis > 50%, and arterial tear/perforation. The area surrounding soft tissue was examined for the presence of access site hematoma or other fluid collections.

### Statistical analysis

Proportions are presented for binary variables. Mean and/or median with standard deviation (SD) are presented for continuous variables. Since this is an observational study with an emphasis on utility of mandatory DUS at discharge for detecting complication to supplement real-world evidence without a control group, no power calculation was performed.

## Results

**Fig. 1:**
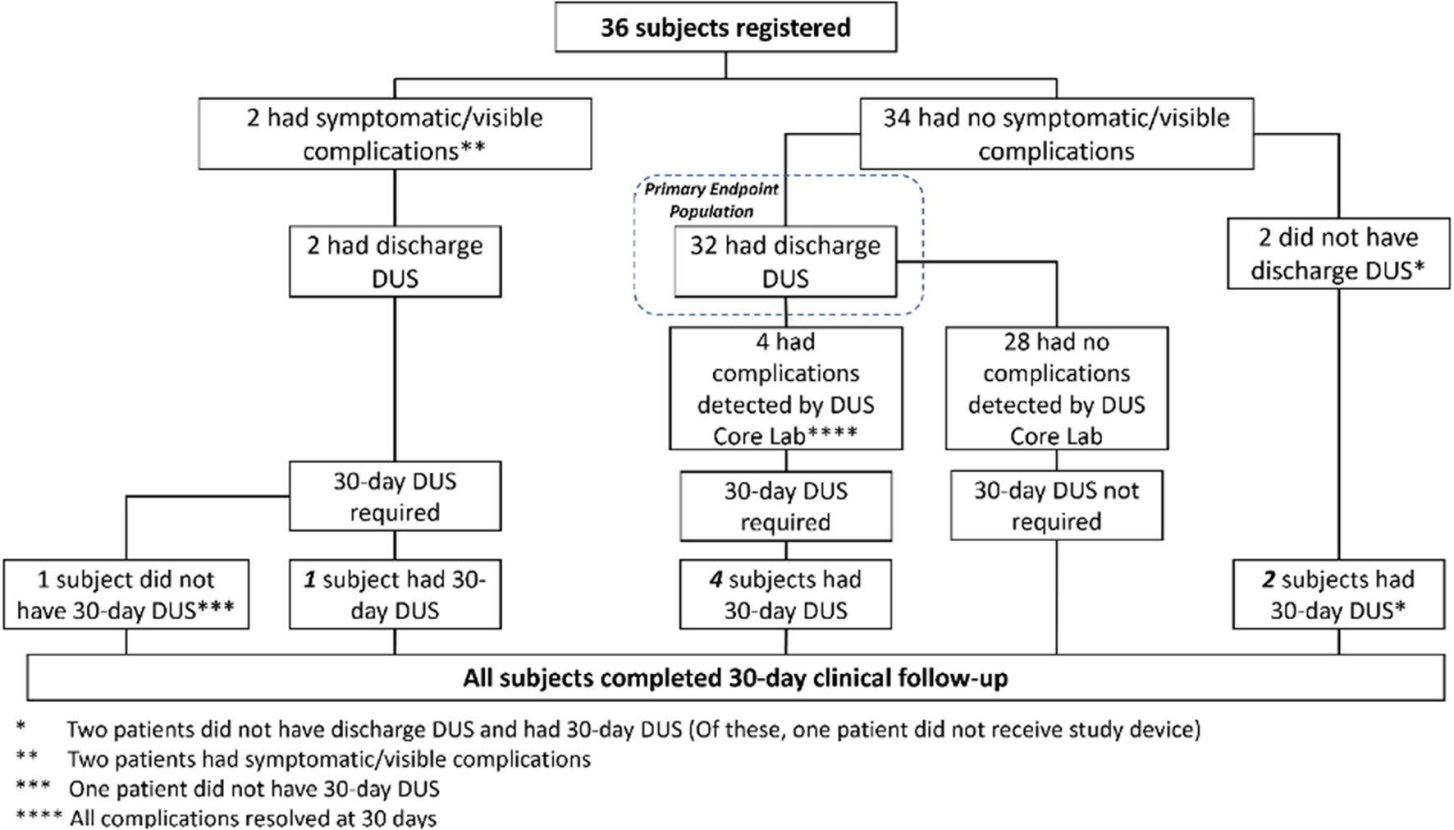
Diagram illustrating patient enrollment, outcomes, and follow-up

Thirty-six consecutive patients who were scheduled to undergo catheter ablation for atrial fibrillation and/or flutter with planned vascular access closure with SMC for all access sites were enrolled (Table 1, Fig. 1). All patients completed 30-day clinical follow-up. The mean age was 62.9 + 10.7 years with 66.7% men. The clinical arrhythmia was paroxysmal atrial fibrillation in 47.2%, persistent atrial fibrillation in 30.6%, and atrial flutter in 16.7%. The majority of patients (91.7%) were on oral anticoagulation at baseline with apixaban used in 86.1% and rivaroxaban in 5.6%. In addition, some patients were on antiplatelet agents, with 8.3% on ASA, 2.8% on clopidogrel, and 2.8% on ticagrelor. The ablation procedure included cryoablation in 38.9% and radiofrequency ablation in 58.3% with one patient having both cryoablation and radiofrequency ablation in a single procedure.

**Table 1:** Baseline patient and procedure characteristics

Characteristic	SMC cohort (*n* = 36)
Age (years)	62.9 ± 10.7
Male	66.7% (24/36)
BMI	31.25 ± 6.39
Diabetes mellitus	33.3% (12/36)
Hypertension	61.1% (22/36)
Dyslipidemia	52.8% (19/36)
Coronary artery disease	30.6% (11/36)
Chronic kidney disease	13.9% (5/36)
Congestive heart failure	11.1% (4/36)
Cerebrovascular accidents	2.8% (1/36)
Paroxysmal atrial fibrillation	47.2% (17/36)
Persistent atrial fibrillation	30.6% (11/36)
Atrial flutter	16.7% (6/36)
Oral anticoagulant at baseline	91.7% (33/36)
Apixaban	86.1% (31/36)
Rivaroxaban	5.6% (2/36)
Oral antiplatelet at baseline	11.1% (4/36)
Aspirin	8.3% (3/36)
Clopidogrel	2.8% (1/36)
Ticagrelor	2.8% (1/36)
Type of ablation procedure	
Cryoablation only	38.9% (14/36)
Radiofrequency ablation only	58.3% (21/36)
Both	2.8% (1/36)
Access legs	
Right only	44.4% (16/36)
Both left and right	55.6% (20/36)
Heparin usage and reversal	
Heparin used	94.4% (34/36)
Heparin reversal with protamine	79% (27/34)

The median number of vascular access sites per patient was 4 with a range from 2 to 5 access sites (Table 2). Access was obtained in the right leg only in 44.4% of patients and bilateral in 55.6%. The distribution of access sites was most commonly 3 access sites on one leg and 1 access site on the contralateral leg in 36.1% of patients, followed by 3 access sites in the right leg only in 30.6%. In 13.9% of patients, there were 2 access sites in the right leg only. A median of 4 SMC devices was used per patient. The success rate of SMC closure in all access sites was 99.2% (120/121). One device failed to deploy properly due to improper technique. The range of access sites closed were 6 to 16Fr with 49.2% being less than 8Fr in size and 37.3% being 8.5–11 Fr. There were 11.9% of sheaths in the 12–14Fr range. The majority of sheaths were closed with a single SMC device (84.9%) with 11.1% requiring two SMC devices per access site. On a per-vein basis, a median of 2 sheaths per vein and 2 SMC devices per vein (*n* = 56) were used, with the majority of procedures using 2 (46.4%) or 3 (46.4%) SMC units per vein. For sheaths greater than 8Fr in size, 21.9% required 2 SMC and 73.4% required 1 SMC device. Mean time to hemostasis was 3.1 + 7.3 min. Median time to ambulation was 193.5 min, and the time to discharge was 5.95 h.

**Table 2:** Access site and closure details

Characteristic	SMC cohort (*n* = 36)
Median number of access sites per patient	4.0 (range 2–5)
Number of access sites per vein	2.3 ± 0.8
Access site distribution per leg	
3 in right leg, 1 in left leg	36.1% (13/36)
3 in right leg	30.6% (11/36)
2 in right leg	13.9% (5/36)
2 in right leg, 2 in left leg	11.1% (4/36)
3 in right leg, 2 in left leg	8.3% (3/36)
Sheath sizes used	
> 15Fr	1.6%
12–14Fr	11.9%
8.5–11Fr	37.3%
< 8Fr	49.2%
No. of SMC used per access site	
1 device	84.9%
2 devices	11.1%
No. of SMC use per access site > 8Fr	
1 device	73.4%
2 devices	21.9%
Post-procedure metrics	
Success rate	99.2% (120/121)
Mean time to hemostasis (minutes)	3.1 ± 7.3
Median time to ambulation (minutes)	193.5
Median time to discharge (hour)	5.95

Out of 36 total patients, 2 were symptomatic or had clinically apparent access site related venous bleeding that required brief additional manual compression to achieve hemostasis. Both received a DUS at discharge, and one out of the two underwent a repeat DUS at 30 days. The other patient did not complete 30-day DUS but had complete resolution of symptoms at clinical follow-up. Of the remainder of the patients, 34 were asymptomatic without any visible evidence of complications. Of these, 32 patients underwent DUS testing at discharge (Fig. 1). The other 2 patients both had a 30-day DUS that was normal. Of the 32 asymptomatic patients, 4 were noted to have abnormal findings on index ultrasound and had repeat DUS scheduled at 30 days.

The overall adverse event rate was 16.7% for both asymptomatic and symptomatic complications (Table 3). Two patients (5.6%) had vascular access site bleeding, and two patients (5.6%) had a hematoma, as adjudicated by CEC. Four patients (11.1%) had a DVT (1 symptomatic and 3 asymptomatic and identified on DUS). There were no major complications detected by DUS. Minor complications identified by DUS only occurred in 12.5% (4/32) of patients, consisting of 3 DVT and 1 hematoma. On the 30-day repeat DUS, all complications had resolved. The patients with DVT were managed by continuing them on the same dose of DOAC or warfarin; they had been on prior to procedure—there was no change in dosage or duration of therapy. There were no additional complications detected at 30-day clinical follow-up.

**Table 3:** Major and minor complications

Complications	SMC (*n* = 36)
Major complications	0.0%
Minor complications (symptomatic + asymptomatic)	16.7% (6/36)*
Deep vein thrombosis	11.1% (4/36)
Vascular access site bleeding	5.6% (2/36)
Hematoma	5.6% (2/36)
Minor complications identified by DUS only	12.5% (4/32)
Deep venous thrombosis	9.4% (3/32)
Hematoma	3.1% (1/32)

^*^One patient had both a DVT and access site bleeding; another patient had access site bleeding and hematoma

The DUS-detected minor complication rate for patients with 2–3 access sites was 7.1% (1/14) and that for patients with 4 or more access sites was 16.7% (3/18). The complication rate was not higher in patients who received 2 SMC per access site as compared to the patients who just received 1 SMC per access site. Among patients who had minor complications, there was no correlation with the number of access sites, protamine use, size of sheaths used, or patient comorbidities.

While statistical analyses were pre-specified for subgroups based on gender, diabetes, and age, there were no major complications detected by DUS in any asymptomatic subjects, which precluded any meaningful analyses of major complications by subgroups.

## Discussion

For patients undergoing a catheter ablation procedure, vascular access site complications have the potential to significantly impact recovery [11–14]. Improvements in vascular access technique, specifically the use of vascular access ultrasound, have significantly reduced complication rates [15–20]. The evolution of post-procedure vascular access site management from manual compression to figure-of-8 stitch or vascular closure devices has also resulted in decreased time to discharge and improved patient satisfaction with the recovery process [21].

This study contributes to an improved understanding of the safety of multi-site suture-mediated vascular access closure in a single vein. The original approval for SMC for venous access was based on single-point, large-bore venous access with a 24F sheath that was used during transcatheter mitral valve repair [6]. While a single point of venous access is typically utilized in structural cardiology and interventional radiology procedures, multiple points of venous access are the norm during electrophysiologic procedures. There has been some concern that the use of multiple SMC devices in adjacent access sites in a single vein might result in a higher rate of complications. Prior studies have demonstrated a low rate of symptomatic clinical endpoints from SMC mediated multi-site venous access following catheter ablation; however, this is the first trial to the authors’ knowledge to utilize scheduled pre-discharge DUS with planned 30 day follow-up DUS for all patients with abnormal findings on index ultrasound.

The results of this trial revealed no major complications and a low rate of clinically evident minor complications in patients undergoing multi-site SMC for venous access. While it is notable that there was a 16.7% rate of minor complications including DVT, access site hemorrhage, and hematoma, it is reassuring that all of these complications were subclinical and resolved at 30 days. It remains unclear if the venous thromboses that were detected by DUS were a result of the expected trauma of access and indwelling sheaths and catheters, or as a direct result of SMC, per se. In the 11.1% of patients that developed a subclinical DVT despite systemic anticoagulation, there was no significant trend in terms of age, gender, number of access sites, size of sheath, protamine use, or other clinical variable. The absence of a control group in this study makes it difficult to determine if this is simply a result of placing sheaths and catheters in the vascular system and could occur in any patient, regardless of closure device or if there is some inherent increase risk with use of a closure device. It is reassuring that all DVT resolved within 30 days on systemic anticoagulation without requiring additional intervention.

The results of this study support the feasibility of multi-site SMC in this patient population. Our data also suggest that DUS is only necessary to evaluate for symptomatic complications, which is consistent with the current standard of care. This study adds to a growing body of literature supporting the safety of vascular closure devices following catheter ablation procedures. While multiple studies have now demonstrated the efficacy of vascular closure in the context of both large-bore and multiple points of venous access [4, 5, 7–9], there are a paucity of data evaluating for potentially silent complications, as well as their natural history. Given the potential for closure devices to improve time to discharge, facilitate same day discharge, and overall improve the patient experience with the recovery process, a more complete understanding of potential safety issues is paramount [5, 7, 8]. This study demonstrates and further supports the relative safety of SMC use in electrophysiology procedures.

## Limitations

Our study had several limitations. First, we utilized a small sample which may have hindered our ability to detect very rare complications. Despite this, our complication rates and distribution were in line with prior studies of vascular closure. Second, our study utilized a non-randomized observational trial design. This limited our ability to determine if there was a causal relationship between SMC and the detected complications. For example, without a non-SMC control group, it remains unclear if the presence of post-procedure DVTs were related to aspects of the procedure (e.g., vascular access, sheath, and catheter insertion) or SMC. However, the goal of this study was designed to be largely descriptive of complications associated with SMC that were potentially clinically important. As such, we felt that prospectively observed serial cohort of all patients undergoing SMC closely mirrored, and was more generalizable, to real-world practice. Moreover, the complication rate observed in this trial, in addition to safety data in the existing literature [5, 7, 8], did not render a signal suggestive of a safety concern that would necessitate a large, randomized controlled trial. A third limitation is the fact that our study was not blinded to clinicians or patients. To address this, our findings were adjudicated by an independent core lab CEC to minimize bias, which we felt was sufficient given the descriptive (as opposed to comparative) nature of the study.

## Conclusion

The use of multi-site SMC of vascular access sites following catheter ablation appears to be safe with no major complications, as well as a low rate of minor complications that completely resolved during follow-up. This includes access sites with both single and 2 or more suture-mediated closures per access site.

## Data Availability

Data are available upon written request to corresponding author.

## Abbreviations

SMC: Suture-mediated closure
DUS: Duplex ultrasound
CEC: Clinical events committee
DVT: Deep venous thrombosis
